# Indications and limits of clear aligner therapy: an international modified Delphi consensus study

**DOI:** 10.1186/s40510-025-00575-1

**Published:** 2025-08-04

**Authors:** Vincenzo D’Antò, Giorgio Oliva, Michele Nieri, Tommaso Clauser, Maria Denisa Statie, Ludovica Nucci, Silvia Caruso, Tommaso Castroflorio, Stephen Chang, Simon Graf, Vincenzo Grassia, Julia Haubrich, Thor Henrikson, Luis Huanca Ghislanzoni, John Kosei Kaku, Kasper Dahl Kristensen, Roberta Lione, Javier Lozano, Björn Ludwig, James Mah, Maurice Meade, Pedro Costa Monteiro, Jean-Marc Retrouvey, Waddah Sabouni, Jörg Schwarze, Bernardo Souki, Flavio Uribe, Nikhillesh Vaiid, Tony Weir, Lorenzo Franchi

**Affiliations:** 1 University of Naples Federico II, Naples, Italy; 2 University of Florence, Florence, Italy; 3 University of Campania Luigi Vanvitelli, Naples, Italy; 4 University of L’Aquila, L’Aquila, Italy; 5 University of Genoa, Genoa, Italy; 6 Private Practice, Taipei, Taiwan; 7 Private Practice, Belp, Switzerland; 8 Private Practice, Cologne, Germany; 9 University of Malmö, Malmö, Sweden; 10 University of Geneva, Geneva, Switzerland; 11 Private Practice, Tokyo, Japan; 12 Aarhus University, Aarhus, Denmark; 13 UniCamillus International Medical University in Rome, Rome, Italy; 14 Private Practice, Murcia, Spain; 15 Homburg University, Homburg, Germany; 16 University of Nevada, Las Vegas, USA; 17 Adelaide Dental School, University of Adelaide, Adelaide, Australia; 18 Private Practice, Porto, Portugal; 19 Baylor College of Medicine, Houston, USA; 20 Saint Antonio University Murcia, Murcia, Spain; 21 Pontifical Catholic University of Minas Gerais, Belo Horizonte, Brazil; 22 University of Connecticut School of Dental Medicine, Farmington, USA; 23 Saveetha Institute of Medical and Technical Sciences, Chennai, India

## Abstract

**Background:**

The aims of this study were to gather expert agreement about essential aspects of clear aligner therapy (CAT) and to determine what research areas need further investigation.

**Materials and methods:**

A steering committee performed literature selection and compiled a list of 25 statements. This study used a modified Delphi method involving a panel of 23 international orthodontic experts. Six essential areas of CAT were investigated: treatment efficacy, quality of life, side effects, management of growing patients, treatment with extraction, and treatment of periodontal patients. A panel of experts assessed 25 statements using a 5-point Likert scale throughout 3 rounds of the study. A steering committee adjusted statements that failed to achieve consensus through either revision, splitting, merging, or complete removal.

**Results:**

After the third round, 22 statements achieved consensus while 3 statements were rejected. The panel agreed that aligners could be used effectively in some types of malocclusions, such as those with mild or moderate crowding or open bite cases. The experts reached a consensus on the biomechanical limits of clear aligners. However, they agreed on the benefits in terms of improved quality of life during treatment and easier maintenance of oral hygiene maneuvers. Regarding specific patient categories, the panelists supported the use of aligners in periodontal patients with tooth migration requiring tipping movements. They also agreed on the advantages of using a rapid palatal expander over aligners in growing patients.

**Conclusions:**

The panel members reached agreement on most topics. However, they acknowledged limitations in the current literature regarding root resorption and orthodontic relapse with CAT compared to fixed appliances. The absence of agreement on treatment duration, effects on skeletal growth, and the management of periodontally compromised patients highlights significant evidence gaps that warrant further research.

**Supplementary Information:**

The online version contains supplementary material available at 10.1186/s40510-025-00575-1.

## Introduction

Clear aligner therapy (CAT) has emerged rapidly as a widely adopted orthodontic treatment modality, with an increasing number of clinicians using it as an alternative to conventional fixed appliances [1, 2]. As CAT is applied to more complex malocclusions, studies have reported low accuracy in achieving certain planned tooth movements [3] suggesting that aligners may not fully replicate the biomechanical control provided by fixed appliances across all types of movements [4]. Despite these limitations, the increasing use of CAT has led researchers to study its effectiveness and limitations through multiple studies and different methodologies. Numerous studies have compared CAT with fixed appliances in terms of treatment outcomes and duration [3–8]​​. In addition, research has explored patient-centered outcomes, including oral health-related quality of life, pain perception, and daily-life impacts during and after treatment [9–11]. Potential side effects also have been explored [10, 12–16], with some evidence suggesting that CAT may result in lower rates of specific adverse effects—such as apical root resorption—compared to fixed appliances. Further investigations have focused on particular patient subgroups, such as growing patients [17–22], those requiring extractions [23–27], and those with periodontal disease [28, 29]. Given the rising popularity of CAT and the breadth of ongoing research, there remains a need to define clearly both its clinical applications and limitations in routine practice. To date, no universal guidelines or consensus have been established to delineate which orthodontic cases are best suited for CAT and which require caution or alternative approaches. Therefore, the present study aimed to reach an expert consensus on the clinical indications and limitations of CAT. Through a modified Delphi methodology, this international initiative seeks to guide clinicians on when and how CAT can be effectively employed, while identifying scenarios where its predictability or efficacy may be reduced. In doing so, this study aims to offer clarity on the role of clear aligners in contemporary orthodontics and to identify future areas of research.

## Materials and methods

### Chair, steering committee, and panelists

This clinical consensus paper was prepared following the ACCORD guidelines [30]. The study was not registered. The consensus process was overseen by LF, who first collaborated with two methodology experts, MN, and TC to define the study protocol. Once the main topic was selected, six subtopics were identified. Two experts, VD and GO, were chosen to form the research team.

The steering committee was composed of:

- Chair: LF.
- Methodology Experts: MN and TC.
- Research Team: VD and GO.
- Liaisons: MDS and LN who assisted in the coordination between the steering committee and the panelists.

Throughout the consensus procedure, the steering committee held online meetings. In cases of disagreement, the decision was taken by the chair. LF identified 30 potential panelists for this study, selecting orthodontists who were authors of peer-reviewed publications and/or international speakers on CAT, with no restrictions on country of origin. The number of panelists was determined to ensure that consensus could be rejected only if more than 2 experts strongly disagreed. As a result, at least 21 participants were required, with rejection rate of less than 30%. Potential panelists were invited to participate by email by the chair. No funding or reimbursement was provided to any participant.

### Literature review and statement formulation

The research team developed specific search strings—as presented in Table 1—using the PICOS model (Population, Intervention, Comparison, Outcome). The subtopics addressed were:

- Efficacy and efficiency of clear aligners.
- Impact of clear aligner treatment on oral health-related quality of life.
- Side effects of CAT.
- Clear aligners in growing patients.
- Clear aligners in extraction treatments.
- Clear aligners and periodontal health.

**Table 1:** Research strings for the topics of the consensus on Pubmed

Research group	Research string
Efficacy and efficiency of clear aligners	(“clear aligne*“[Title/Abstract] OR “orthodontic aligne*“[Title/Abstract] OR Invisalign[Title/Abstract]) AND ((“treatment outcome“[MeSH Terms]) OR (“effect*“[All Fields] OR “effic*“[All Fields] OR “accurac*“[All Fields])) AND (2018:2024[pdat]) AND (“clinical trial“[Publication Type] OR “systematic review“[Publication Type])
Quality of life and clear aligner treatment	(“clear aligne*“[Title/Abstract] OR “orthodontic aligne*“[Title/Abstract] OR Invisalign[Title/Abstract]) AND (“quality of life“[MeSH Terms]) AND (2018:2024[pdat]) AND (“clinical trial“[Publication Type] OR “systematic review“[Publication Type])
Side effects of clear aligner treatment	(“clear aligne*“[Title/Abstract] OR “orthodontic aligne*“[Title/Abstract] OR Invisalign[Title/Abstract]) AND (“side effect*“[Title/Abstract] OR “adverse effect*” OR “speech“[Title/Abstract] OR “root resorption“[Title/Abstract] OR “pain“[Title/Abstract]) AND (2014:2024[pdat])
Clear aligners in growing patients	(“clear aligne*“[Title/Abstract] OR “orthodontic aligne*“[Title/Abstract] OR Invisalign[Title/Abstract]) AND (“orthopedic“[Title/Abstract] OR “functional“[Title/Abstract] OR “advancement“[Title/Abstract] OR “Class II“[Title/Abstract] OR “Class two“[Title/Abstract] or “Class III“[Title/Abstract] OR “Class three“[Title/Abstract]) AND (2014:2024[pdat])
Clear aligners in extraction treatments	(“clear aligne*“[Title/Abstract] OR “orthodontic aligne*“[Title/Abstract] OR Invisalign[Title/Abstract]) AND (“extraction*”) AND (2014:2024[pdat])
Clear aligners and periodontal health	(“clear aligne*“[Title/Abstract] OR “orthodontic aligne*“[Title/Abstract] OR Invisalign[Title/Abstract]) AND (periodontal disease[MeSH Terms] OR “periodontal“[Title/Abstract]) AND (2014:2024[pdat])
The research was performed on August 10th, 2024	

Systematic reviews and randomized controlled trials were given priority, and if they were not available, the teams would use prospective or retrospective non-randomized clinical studies.

This literature review had two primary objectives:

1. To identify existing evidence gaps to be addressed during the consensus conference.
2. To select at least one high-quality study per subtopic (about 15 in total) to be shared with the panelists at a later stage.

This phase included multiple meetings of the steering committee to address methodological challenges and finalize the selected studies. After gathering the literature, the research team drafted an initial list of statements, which was subsequently refined in a full steering committee meeting. Each statement was designed to be concise and easily understandable to facilitate meaningful feedback from the panelists.

## Consensus rounds

The process was pre-planned to include a maximum of four rounds. Every round had three stages which included voting, consensus evaluation, and final statement decision.

### Voting

Each panelist was given a unique ID by RStudio (RStudio Team, 2020). A four-character substring that was extracted from a Universally Unique Identifier (UUID) was checked for uniqueness and then given to each participant. Only MDS and LN had access to these codes during the process, and the steering committee remained blinded to individual identities. Voting was conducted via Google Docs Forms survey, and panelists were required to input their anonymous codes without providing personal information (e.g., name, email). A 5-point Likert scale was used by the panelists to rate each statement, as presented in Table 2. Panelists who selected “neutral,” “disagree,” or “strongly disagree” were required to provide a comment that could be used to improve the statement in the next round. The responses were to be submitted within a period of one to three weeks, depending on the expected workload. If a panelist did not respond, MDS and LN would identify the missing code, and the chair would send a reminder. Anonymity was maintained throughout the process; final reports did not include the order or timing of responses. Panelists who failed to respond after three reminders were excluded from subsequent rounds.

**Table 2:** The likert scale items and their meaning

Likert value	Meaning
1	Strongly disagree
2	Disagree
3	Neutral
4	Agree
5	Strongly agree

### Consensus evaluation

To meet the criteria for consensus, each statement had to satisfy all of the following:

1. At least 70% of panelists agreed or strongly agreed with the statement.
2. Fewer than 20% of panelists disagreed or strongly disagreed.
3. Fewer than 10% strongly disagreed.

These criteria were established prior to the start of the study and remained unchanged. The research teams independently evaluated the voting results before submitting their initial recommendations to the full steering committee. If disagreements persisted, the chair had the authority to make the final decision—although this scenario did not occur.

### Statement decision

Following consensus evaluation, the research teams proposed decisions for each statement, that were reviewed by the full steering committee. When necessary, the chair was empowered to make the final determination; however, this was not required. Statements that reached consensus were accepted and excluded from subsequent voting rounds.

For statements not reaching consensus, three alternatives were considered:

1. Rephrased: The statement received possible modifications to increase its chances of gaining more extensive approval during the subsequent voting period.
2. Rejected: When the steering committee recognized that achieving consensus was impossible because members held different positions, the statement was eliminated from future voting.
3. Split/Merged: The committee made adjustments by splitting statements into two parts or merging them with another statement when they believed this approach would create a more acceptable consensus; however, this approach was avoided, when possible, to preserve clarity.

After the steering committee finished their decision-making process for individual statements, they developed an updated survey containing rephrased and split or merged statements.

## Results

A steering committee meeting was first convened in June 2024. The evidence selection was due for completion and transmission by the end of October 2024. Some update meetings were planned for July, September, and October 2024. Meanwhile, the chair sent invitations to potential panelists. Twenty-three individuals accepted the invitation and were subsequently included in the panel (Table 3).

**Table 3:** Panelists included in the Delphi study

Name of the panelist	Affiliation
Silvia Caruso	Department of Life, Health and Environmental Science, University of L’Aquila, L’Aquila, Italy
Tommaso Castroflorio	Department of Sciences Integrated Surgical and Diagnostic, University of Genoa, Genoa, Italy
Stephen Chang	Private Practice, Taipei, Taiwan
Simon Graf	Private Practice, Belp, Switzerland
Vincenzo Grassia	Multidisciplinary Department of Medical, Surgical, and Dental Specialties, University of Campania Luigi Vanvitelli, Napoli, Italy
Julia Haubrich	Private Practice, Köln, Germany
Thor Henrikson	Department of Orthodontics, University of Malmö, Malmö, Sweden
Luis Huanca Ghislanzoni	Department of Orofacial Rehabilitation, University of Geneva, Geneva, Switzerland
John Kosei Kaku	Private Practice, Tokyo, Japan
Kasper Dahl Kristensen	Section of Orthodontics, Department of Dentistry and Oral Health, Aarhus University, Aarhus, Denmark
Roberta Lione	UniCamillus International Medical University in Rome, Rome, Italy
Javier Lozano	Private Practice, Murcia, Spain
Björn Ludwig	Homburg University, Homburg, Germany
James Mah	School of Dental Medicine, University of Nevada, Las Vegas, USA
Maurice Meade	Adelaide Dental School​, University of Adelaide, Adelaide, South Australia
Pedro Costa Monteiro	Private Practice, Porto, Portugal
Jean-Marc Retrouvey	School of Dental Medicine, Department of Molecular Genetics, Baylor College of Medicine, Houston, USA
Waddah Sabouni	Saint Antonio University Murcia, Murcia, Spain
Jörg Schwarze	Private Practice, Cologne, Germany
Bernardo Souki	School of Dentistry, Graduate Program in Orthodontics, Pontifical Catholic University of Minas Gerais, Belo Horizonte, Brazil
Flavio Uribe	Department of Orthodontics, University of Connecticut School of Dental Medicine, Farmington, USA
Nikhillesh Vaiid	Saveetha Institute of Medical and Technical Sciences, Chennai, India
Tony Weir	Adelaide Dental School, University of Adelaide, Adelaide, South Australia

The steering committee met in November 2024 to confirm the final selection of 15 papers [3–5, 9, 12–14, 18–24, 29] (Supplementary Table). During the same meeting, the topics for the statements were also listed. The papers were sent to the panelists on November 23, 2024. The steering committee met on December 23, 2024, to finalize the list of 25 statements. The efficacy and efficiency of clear aligners subtopic received 11 statements, quality of life and clear aligner treatment received 3 statements, side effects of clear aligner treatment received 3 statements, clear aligners in growing patients received 2 statements, clear aligners in extraction treatments received 3 statements, and clear aligners and periodontal health received 3 statements.

The first round of consensus started on December 23, 2024, and the voting phase ended on January 13, 2025. The response rate of the panelists was 100%. Consensus was achieved for 10 of the 25 statements: 2 on the efficacy and efficiency of clear aligners, 2 on the quality of life and clear aligner treatment, 1 on the side effects of clear aligner treatment, 1 on clear aligners and growing patients, 2 on clear aligners and extraction treatments, and 2 on clear aligners and periodontal health. On January 23, 2025, the steering committee met to define a new list of statements. All of the 10 statements that achieved consensus were accepted. All of the 15 statements which failed to achieve consensus were revised and forwarded to the second round.

The second round started on January 24, 2025, and was due to end on February 2. The voting phase was extended and closed on February 10, 2025, after follow-up reminders were sent to the late panelists. The response rate of the panelists was 100%. Seven statements reached consensus, while 8 did not. The steering committee met on May 23, 2025, to finalize a new list of statements. The 7 statements that reached consensus were accepted. Two of the 8 statements that did not achieve consensus were rejected, while 6 were rephrased. Hence, 6 statements were forwarded to the third round.

The third round began on February 23, 2025, with a due date set for March 10. The voting phase closed on March 17, 2025, after the panelists who were late were sent reminders. The response rate of the panelists was 100%. Five out of the 6 statements reached a consensus. The statement that did not achieve consensus was rejected.

The fourth round was not necessary.

## Consensus

The consensus conference participants established strong agreements about multiple points. The final statements are reported in Table 4.

**Table 4:** Statements results

#	Statement	Strongly disagreed (%)	Disagree (%)	Agree and strongly agree (%)	Consensus	Last round of appearence
	*Efficacy and efficiency of clear aligners*					
1	For Class I non-extraction compliant patients with mild to moderate crowding, normal overjet and overbite, and rotations smaller than 20 degrees, treatment duration is shorter with clear aligners than with conventional fixed appliances	4	13	65	None	3rd
2	Treatment with clear aligners is an effective alternative to conventional fixed appliances in Class I non-extraction compliant patients with mild to moderate crowding	0	4	91	Consensus	3rd
3	Clear aligners can be changed every 7 to 14 days depending on patient compliance, periodontal conditions, amount of tooth movement, and aligner material	0	4	87	Consensus	2nd
4	Treatment with clear aligners is an effective alternative to conventional fixed appliances for managing open bite cases	0	4	87	Consensus	3rd
5	Expansion with clear aligners is achieved primarily through crown tipping rather than bodily tooth movement	0	0	96	Consensus	3rd
6	Clear aligners can distalize the upper first molar by up to 2.5 mm, considering bodily movement, distal inclination, and rotational correction	0	9	74	Consensus	2nd
7	Clear aligners often fail to fully correct premolar and canine angulation, inclination, and rotation, requiring auxiliaries, refinements, or overcorrection	0	9	91	Consensus	3rd
8	Orthodontic relapse is similar in cases treated with clear aligners and in those treated with conventional fixed appliances, although the level of evidence is low	0	4	78	Consensus	3rd
9	Planning unpredictable movements with clear aligners increases the likelihood of additional refinements.	0	4	91	Consensus	1st
10	Planning unpredictable movements with clear aligners increases the likelihood of switching to conventional fixed appliances	4	4	83	Consensus	1st
11	Clear aligner treatment is more expensive but requires less chair time for the orthodontist compared to conventional fixed appliances	4	4	87	Consensus	2nd
	*Impact of clear aligner treatment on oral health-related quality of life*					
12	Oral health-related quality of life is better in patients treated with clear aligners compared to those treated with conventional fixed appliances	4	4	83	Consensus	1st
13	There are no differences in oral health-related quality of life at the end of treatment between patients treated with clear aligners and those treated with conventional fixed appliances, although the level of evidence is low	4	9	87	Consensus	2nd
14	Patients treated with clear aligners experience less pain during treatment compared to those with conventional fixed appliances	0	0	83	Consensus	1st
	*Side effects of clear aligner treatment*					
15	Treatment with clear aligners commonly causes temporary phonetic issues for most patients, which typically resolve within a few days	0	0	100	Consensus	2nd
16	The degree of root resorption is lower with clear aligners compared to conventional fixed appliances, although the level of evidence is low	0	13	78	Consensus	2nd
17	White spots and carious lesions are less frequent in patients treated with clear aligners compared to those treated with conventional fixed appliances	4	9	97	Consensus	1st
	*Clear aligners in growing patients*					
18	Treatment with a rapid palatal expander produces more skeletal expansion with less dental tipping than treatment with clear aligners	0	0	96	Consensus	1st
19	There are no differences in the skeletal changes achieved in Class II patients treated with either clear aligners featuring a mandibular advancement device or traditional fixed and removable functional appliances, although the level of evidence is low	9	4	69.6	None	2nd
	*Clear aligners and extraction treatments*					
20	In patients with Class I molar relationships and severe crowding, treatments requiring the extraction of four first premolars achieve less favorable alignment outcomes with clear aligners compared to conventional fixed appliances	0	17	78	Consensus	2nd
21	In patients with Class I molar relationships and severe crowding, treatments requiring the extraction of four first premolars result in fewer occlusal contacts when using clear aligners compared to conventional fixed appliances	0	17	78	Consensus	1st
22	In patients with Class I molar relationships and severe crowding, treatments requiring the extraction of four first premolars using clear aligners result in reduced upper incisor torque and greater tipping movements of the upper canines, premolars, and molars at the end of therapy compared to conventional fixed appliances	4	4	87	Consensus	1st
	*Clear aligners and periodontal health*					
23	Patients treated with clear aligners accumulate less plaque and have less gingival bleeding compared to those treated with conventional fixed appliances	0	0	100	Consensus	1st
24	Treatment with clear aligners is not suitable for patients with periodontal disease stability and a reduced periodontium when bodily movements are required	13	43	39	None	2nd
25	Treatment with clear aligners is suitable for correcting dental migration caused by periodontal disease when it can be achieved through tipping movements and relative extrusion.	0	0	96	consensus	1st

### Efficacy and efficiency of clear aligners

Among 11 statements on efficacy and efficiency of clear aligners, experts reached consensus on 10. The panel members agreed that clear aligners achieved the same results as traditional fixed appliances for treating non-extraction Class I cases with mild to moderate crowding (91% agreement). The panel members endorsed protocols that included regular aligner changes at intervals of 7–14 days (87% agreement) and open bite management strategies (87% agreement). The panel members agreed that clear aligners produce dental movements primarily through crown tipping (96% agreement), and they recognized the possibility of upper first molar distalization up to 2.5 mm (74% agreement) while showing strong support for auxiliaries in complex movement management like canine inclination and rotation (91% agreement). The lack of difference in relapse between CAT and conventional fixed appliances found consensus among the panelists (78%, agreement). However, the panelists highlighted a low level of evidence to support this statement. The panelists agreed that planning unpredictable movements increases the likelihood of additional refinements (91% agreement) and increases the likelihood of switching to conventional fixed appliances (83% agreement). Regarding the increased cost of clear aligners, the panel agreed that the reduction in chair time provided sufficient benefit (87% agreement).

### Impact of CAT on oral health-related quality of life

All the 3 statements on oral health-related quality of life reached consensus. The panel members agreed that patients who receive CAT experience better oral health-related quality of life compared to those treated with conventional orthodontic (83% agreement) along with less pain (83% agreement) during treatment but showed no meaningful quality-of-life changes at the end of treatment (87% agreement).

### Side effects of CAT

All the 3 statements on side effects reached consensus. All participants agreed that phonetic problems briefly arise after clear aligner use (100% agreement) but typically resolve within a few days. According to the panelists, clear aligners demonstrate better performance than conventional fixed appliances in two areas: they reduce root resorption rates (78% agreement) and minimize the occurrence of white spots and carious lesions (97% agreement).

### Clear aligners in growing patients

Out of the 2 statements on clear aligners in growing patients only 1 reached consensus. According to the panelists for growing patients, the use of a rapid palatal expander produces greater skeletal expansion with reduced dental tipping than clear aligners (96% agreement).

### Clear aligners and extraction treatment

All the 3 statements on clear aligners and extraction treatment reached consensus. Panelists agreed that in extraction treatments for severe crowding, clear aligners yield less favorable outcomes compared to conventional fixed appliances in terms of alignment outcomes (78% agreement), occlusal contacts (78% agreement) and control of incisor torque and tipping of the upper canines, premolars, and molars (87% agreement).

### Clear aligners and periodontal health

Out of 3 statements on clear aligners and periodontal health, experts found consensus on 2. The panel unanimously agreed (100% agreement) that clear aligners result in less plaque accumulation and gingival bleeding compared to fixed appliances. Clear aligners were favored for managing minor dental migration caused by periodontal disease when it can be achieved through tipping movements and relative extrusion (96% agreement).

## Discussion

This international study used a modified Delphi methodology to determine the proper applications of clear aligner therapy (CAT). The modified Delphi method used in this study follows a proven approach to achieve expert consensus in healthcare research especially when high-quality evidence is scarce or clinical decision-making needs to combine various perspectives. The Delphi method conducts multiple rounds of questioning which differs from traditional survey methods. The selected literature was distributed to panelists one month before starting the consensus process. The researchers followed established Delphi methodology guidelines by providing all experts with the same evidence base to ensure they made decisions based on the same information. The Delphi method aims to produce informed consensus instead of relying on intuitive opinions despite its potential impact on expert opinions. The use of a predetermined scoring and decision-making method, combined with anonymous data collection, ensures consistency in this methodology. The panelists showed agreement on 22 statements.

### Efficacy and efficiency of clear aligners

The panel agreed that clear aligners are as effective as conventional fixed appliances in treating Class I non-extraction cases with mild to moderate crowding (statement #2, Table 4), a finding that is consistent with the current literature [3]. However, no consensus was reached regarding treatment duration (statement #1, Table 4). A systematic review by Alhamwi et al. [5] found no significant difference in overall treatment time for cases involving mild to moderate crowding. This result received a low GRADE recommendation because some studies showed inconsistency while others contained high levels of bias. Among the studies included in the review, five clinical trials reported shorter treatment durations with clear aligners, two favored fixed appliances, and three found no difference. The authors concluded that additional randomized controlled trials are needed to clarify this issue. The panel supported the efficacy of aligner changes every 7–14 days (statement #3, Table 4) and endorsed the use of aligners in open bite management (statement #4, Table 4), both of which are supported by recent clinical research [4, 31]. Regarding open bite treatment, the supporting evidence requires careful interpretation based on the most recent systematic review and meta-analysis by Correa et al. [32]. This comprehensive analysis of 14 studies (12 included in meta-analysis) demonstrated that clear aligner therapy leads to significant anterior open bite correction of approximately 2.76 mm (95% CI: 2.23–3.28), which can be attributed primarily to incisor extrusion rather than molar intrusion. Specifically, the meta-analysis revealed significant maxillary incisor extrusion of 0.85 mm (95% CI: 0.43–1.26) and mandibular incisor extrusion of 0.86 mm (95% CI: 0.29–1.44), while no significant changes were identified for maxillary and mandibular molar intrusion or mandibular plane angle. The panelists almost unanimously agreed (statement #5, Table 4) that arch expansion occur with tipping movements. A recent literature review [33] highlights how the body movements obtained are approximately 50% of those planned in the upper molar area. This result was also supported by a study on CBCT [34]. Agreement was also reached on the potential for molar distalization up to 2.5 mm (statement #6, Table 4) and on the essential role of auxiliaries in managing complex movements such as canine rotation and inclination (statement #7, Table 4). These positions align with the existing literature regarding the biomechanical limitations of CAT [4, 35–39]. Experts agreed that there is no difference in terms of treatment relapse between clear aligners and conventional fixed appliances (statement #8, Table 4). However, they stressed that further studies are needed. A randomized clinical trial with 6-month follow-up reports no difference between the two methods for patients with mild-moderate malocclusions [40]. Retrospective studies with longer follow-ups do not agree on their results [41]. Further studies are needed to evaluate the stability of treatment with clear aligners. The panelists agreed that planning unpredictable movements leads to additional refinements (statements #9, Table 4) and increase the likelihood of switching to fixed appliances (statement #10, Table 4). This is an often overlooked problem of CAT, as highlighted by Kravitz et al. [42] In their research, they indicate that on average 2.5 refinements are needed to complete the therapy, but more than 10% of the cases analyzed had 5 or more refinements with related additional time and costs. One in every 6 of their patients needed to switch to conventional fixed appliances. Despite this the panelists accepted the statement that reduced chair time justifies the higher costs (statement #11, Table 4) even though this topic lacks thorough discussion in the literature [43, 44].

### Impact of CAT on oral health-related quality of life

The panel reached consensus that patients undergoing CAT experience superior oral health-related quality of life (OHRQoL) and reduced pain during the active phase of treatment (statements #12 and #14, Table 4). These findings are supported by the systematic review and meta-analysis conducted by Li et al. [9] that confirmed better OHRQoL outcomes and lower pain perception among CAT patients, particularly in the early stages of treatment. The panel members agreed that OHRQoL results between treatment groups show no significant differences after the therapy (statement #13, Table 4). This consensus was reached during the second voting round, with panelists noting the limited available evidence on long-term comparisons. The panel recommended that future research should stratify findings based on malocclusion complexity and other relevant parameters to develop more precise clinical indications.

### Side effects of CAT

The panel unanimously agreed (statement #15, Table 4) that phonetic disturbances may occur temporarily with the use of clear aligners, a finding corroborated by Ali Baeshen et al. [14]. Consensus was also reached on the reduced risk of root resorption (statement #16, Table 4) and the lower incidence of white spot lesions and carious lesions (statement #17, Table 4) compared to conventional fixed appliances. Panelists, however, highlighted the limited availability of high-quality literature on root resorption in CAT. Zhang and coworkers [12], in a systematic review of systematic reviews on root resorption, found that CAT could result in less severe root resorption than conventional fixed appliances. The four systematic reviews had either low or critically low quality. Most of these systematic reviews did not account for multiple testing or clustering. One of the meta-analyses [45] was affected by a unit of analysis error, in which some studies were included multiple times, leading to artificially inflated sample sizes. There was only one randomized controlled trial (RCT) [46] among all the systematic reviews analyzed by Zhang et al. [12] that concluded that on premolars there are no difference in root resorption between clear aligners and fixed appliance with light forces. Additionally, Yassir et al. [47], and Dawood et al. [48], reported in two systematic reviews that the magnitude of the applied force—rather than the type of appliance—was associated with root resorption, together with several other factors. Based on the current literature, therefore, the evidence regarding root resorption in CAT should be considered inconclusive. The reduced incidence of white spot lesions is further supported by the meta-analysis conducted by Raghavan et al. [13], that attributed this benefit to improved oral hygiene associated with removable aligners. The authors based their results on 5 RCTs yet al.l these studies exhibited high risk of bias. The studies remained vulnerable to errors because blinding was impossible, yet researchers detected data reporting errors and intervention protocol modifications in most RCTs. The panel emphasized that the validation of these findings through prospective clinical trials could enhance the evidence base and support the development of more precise clinical indications for CAT.

### Clear aligners in growing patients

The panel members almost unanimously agreed that rapid palatal expanders produce more significant skeletal expansion and result in less dental tipping compared to clear aligners (statement #18, Table 4). This view is supported by current literature, which indicates that when used for expansion, clear aligners primarily induce dentoalveolar rather than true skeletal changes [20]. However, the panel did not reach consensus on the effectiveness of clear aligners as functional appliances for growing patients with Class II malocclusion due to mandibular retrusion (statement #19, Table 4). Although this population remains a focus of active research, yet the role of CAT in skeletal modifications needs further exploration, particularly when compared to established functional or orthopedic devices. D’Antò [49] and others found that most of the research knowledge of this topic is based on observational studies with problems in methodology, while the few RCT reported show low risk of bias. As stated by Sim and Farella [50], RCTs with proper sample size calculation and allocation are needed for this topic.

### Clear aligners and extraction treatments

The panel members agreed that extraction cases with severe crowding produce worse results with CAT because it affects, alignment (statement #20, Table 4), occlusal contacts (statement #21, Table 4) and incisor torque and tipping of the upper canines, premolars, and molars (statement #22, Table 4). Jaber et al. [23] conducted a study comparing fixed appliances with clear aligners in extraction cases and demonstrated that both approaches produce equivalent outcomes except for occlusal contacts. Song and coworkers [24] explored the variations of tooth movements in extraction cases, highlighting the restricted capabilities of clear aligners to perform bodily movements. Similar results were published by Dai and coworkers [51]. In the future, research should concentrate on complex treatments and the effects of auxiliaries, including mini-screws and hybrid mechanics.

### Clear aligners and periodontal health

The panel unanimously agreed (statement #23, Table 4) that clear aligner therapy (CAT) is associated with reduced plaque accumulation and gingival bleeding compared to fixed appliances. This consensus reflects the clinical observation that removable aligners allow for better oral hygiene maintenance compared to fixed appliances. However, the clinical significance of these differences remains a subject of debate in the literature. While Di Spirito et al. [29] and Llera-Romero et al. [52] suggest in their systematic reviews that the differences in periodontal parameters between clear aligners and fixed appliances may not reach clinical significance thresholds, the panel’s unanimous agreement indicates that practicing clinicians observe meaningful differences in their daily practice. This discrepancy between statistical significance in research studies and clinical relevance in practice may be attributed to several factors, including variations in patient compliance, differences in oral hygiene instruction protocols, and the heterogeneity of study populations in the reviewed literature. The panel’s consensus suggests that the practical benefits of improved oral hygiene with clear aligners, while potentially modest in magnitude, are consistently observed and clinically relevant for patient care. The panel members agreed (statement #25, Table 4) that CAT is the preferred choice for treating minor dental migrations in periodontally compromised patients. However, no consensus was reached regarding its use for major bodily movements in this population (statement #24, Table 4). The panel emphasized the need for further research in this area. Meanwhile, the use of clear aligners in patients with periodontal disease should be guided by careful clinical judgment and a thorough evaluation of individual biomechanical needs.

## Conclusions

The use of clear aligners has become increasingly widespread as an orthodontic treatment modality. The panelists reached consensus on the majority of statements, consistently aligning with the current scientific literature. Notably, agreement was achieved on some topics—such as orthodontic relapse and oral hygiene—even if in the absence of robust supporting evidence. Conversely, no consensus was reached on treatment duration, the skeletal effects of aligners in growing patients, or the comprehensive management of periodontally compromised individuals. These gaps underscore the need for further high-quality research to clarify the clinical indications and limitations of CAT in these domains.

## Supplementary Information

Below is the link to the electronic supplementary material.

Supplementary Material 1

## Data Availability

No datasets were generated or analysed during the current study.
