# Supplementary material for: Indications and limits of clear aligner therapy: an international modified Delphi consensus study
Source: Prog Orthod. 2025 Aug 4;26:28. doi: 10.1186/s40510-025-00575-1 (PMC12321729; doi:10.1186/s40510-025-00575-1)
Supplement: Supplementary file 1 — Supplementary Material 1 [file 40510_2025_575_MOESM1_ESM.docx]

**Supplementary Table**: List of papers provided to the panelists.

| **Study** | **Topic** | **Type of study** | **Aim** |
| --- | --- | --- | --- |
| Alhamwi et al. 2024 [5] | Efficacy and efficiency of clear aligners | Systematic review | To critically appraise and assess the currently observed evidence about the difference in orthodontic treatment duration between clear aligners and fixed appliances in crowding cases. |
| Alwafi et al. 2023[3] | Efficacy and efficiency of clear aligners | Systematic review | to systematically assess existing scientific evidence through an overview of SRs to evaluate the predictability of tooth movements with CAT and compare treatment outcomes between CAT and FA. By doing so, we seek to provide a comprehensive appraisal of the current knowledge on this topic. |
| Castroflorio et al. 2022 [4] | Efficacy and efficiency of clear aligners | Prospective observational study | to verify the hypothesis that clear aligner treatment does not completely fulfill the pretreatment goals at the end of the first set of aligners. |
| Li et al. 2023 [9] | Quality of life and clear aligner treatment | Systematic review | to compare the pain intensity and impacts on oral health-related quality of life between orthodontic patients treated with clear aligners and fixed appliances. |
| Ali Baeshen et al. 2023 [4] | Side effects of clear aligner treatment | Systematic review | To assess the effect of clear aligners on the speech of patients undergoing orthodontic therapy through a systematic review of the literature. |
| Raghavan et al. 2023 [13] | Side effects of clear aligner treatment | Systematic review | To analyse the available evidence regarding the incidence and severity of white spot lesions, plaque accumulation and salivary caries-associated bacteria in clear aligners verses conventional fixed orthodontic appliances. |
| Zhang et al. 2024 [12] | Side effects of clear aligner treatment | Systematic review | To evaluate the current evidence on clear aligners and root resorption using 3D and/or combined 2D and 3D methods from available systematic reviews and meta-analyses and to determine the relationship between root resorption and clear aligners using the AMSTAR 2 tool. |
| Bruni et al. 2024 [20] | Clear aligners and growing patients | Randomized controlled trial | to evaluate the efficacy of Invisalign First Phase I treatment compared with toothborne rapid maxillary expansion (RME) in mixed dentition patients by examining changes in palatal volume, palatal surface area, and maxillary interdental transverse measurements. |
| Hosseini et al. 2023 [19] | Clear aligners and growing patients | retrospective controlled study | to compare the skeletal and dental changes of patients with a Class II relationship treated with clear aligner mandibular advancement (MA) and Herbst appliances followed by comprehensive orthodontic treatment. |
| Lombardo et al. 2024 [18] | Clear aligners and growing patients | retrospective controlled study | to compare the changes produced by the Twin block versus those by Mandibular Advancement. |
| Meade and Weir 2023 [22] | Clear aligners and growing patients | retrospective controlled study | to determine whether the achieved changes in the treatment of Class II malocclusion were the same as those planned after the prescribed wear of an initial phase of Invisalign treatment with the mandibular advancement appliance (MAA) |
| Zybutz et al. 2021 [21] | Clear aligners and growing patients | cross-sectional observational survey | To compare patients’ experiences with the Invisalign Teen with Mandibular Advancement (ITMA) and Twin Block (TB) appliances, both initially and after several months of wear. |
| Jaber et al. 2023 [23] | Clear aligners and extraction treatments | Randomized controlled trial | To compare the effectiveness of the clear aligners with the traditional fixed appliances in the treatment of premolars extraction complex cases using the American Board of Orthodontics Objective Grading System (ABO-OGS). |
| Song et al. 2024 [24] | Clear aligners and extraction treatments | retrospective controlled study | to compare the results of tooth movements after maximum retraction was performed with Invisalign and fixed appliances on the patients with the 4 first premolars extracted |
| Di Spirito et al. 2023 [29] | Clear aligners and periodontal health | Systematic review | to summarize the current evidence in order to assess whether clear aligners are associated with a more beneficial impact on periodontal health status compared to fixed appliances in patients undergoing orthodontic treatment. |
